# Quality and Reliability Analysis of Essential Tremor Disease Information on Social Media: The Study of YouTube

**DOI:** 10.5334/tohm.727

**Published:** 2022-11-08

**Authors:** Erman Altunisik, Yasemin Ekmekyapar Firat

**Affiliations:** 1 Department of Neurology, Adiyaman University Faculty of Medicine, Adiyaman, Turkey; 2 Department of Neurology, Sanko University Faculty of Medicine, Gaziantep, Turkey

**Keywords:** Essential tremor, YouTube, DISCERN, information, quality

## Abstract

**Background:**

YouTube has evolved into an important educational tool and information source for patients and their families. YouTube videos have the potential to influence the patients’ health-related diagnosis and treatment decision-making. We aimed to evaluate the quality and usefulness of videos on essential tremor available on YouTube using quantitative instruments.

**Methods:**

Video searches were performed by typing the keywords ‘essential tremor’, ‘postural tremor’, ‘action tremor’, ‘essential tremor hand’, and ‘essential tremor head’ into the YouTube search bar. The top 30 videos were reviewed for each search term. The videos were analyzed by two independent raters using the DISCERN and Global Quality Scale (GQS) scoring systems. Qualitative and quantitative data were recorded for each video.

**Results:**

A total of 83 videos were analyzed. The mean DISCERN score was 41.96 out of 75 possible points, and the mean GQS score was 2.97 out of 5 possible points. Twelve percent of the videos were categorized as very poor, 38.6% as poor, 20.5% as fair, 19.3% as good, and 9.6% as excellent. The videos containing qualitative content features, such as clear information, symptoms, etiology, diagnosis, treatment, treatment response, epidemiology, diagrams, and radiological images had significantly higher DISCERN and GQS scores.

**Discussion:**

We consider that reliable and useful content is not provided on YouTube for individuals searching for information on essential tremor. We listed the best-quality videos on this topic as a reference for healthcare practitioners and patients. Patients should always verify information provided in such videos through more reliable sources.

## Introduction

Health information is one of the most sought-after internet topics on the internet in the USA and Europe [1]. YouTube (YT) is the world’s most widely-used video hosting site; it is the second-most popular website after Google [2]. Many users consider the internet a valuable and reliable source of health-related information; many consult online sources before seeking professional help [3]. This behavior has increased steadily in recent times, especially during the COVID-19 pandemic [4]. YT videos have the potential to influence the decision-making processes of patients and their relatives and affect how they manage their illness and their daily lives [5]. Therefore, as one of the most accessed websites and video-sharing platforms worldwide, YT is likely to spread misleading information to healthcare consumers, which can have devastating effects. It is extremely important to use appropriate tools to test and audit the information that is shared on this platform [6]. Unlike journal articles or guidelines, which are subject to intensive review and detailed evaluation, there is no review process for videos uploaded to YT.

The prevalence of noncommunicable diseases is on the increase worldwide. Among these diseases, neurological disorders, some of which have high global mortality and morbidity rates, occupy an important place in this increase [7]. However, there remains a dearth of studies that analyze the reliability of medical information on social media platforms, especially with regard to chronic neuropsychiatric conditions.

Internationally, the most common movement disorder has been shown to be essential tremor (ET). The symptoms of this disorder occur during voluntary movement and involve a type of tremor that affects the hands and arms at a frequency of 8 to 12 Hz. These tremors may also be accompanied by tremors of the head or voice. While the prevalence of ET sits at 0.9% among all age groups, its prevalence is increasing in individuals over 65 years of age [8]. As there is no specific biomarker, laboratory test, or imaging method for diagnosis of ET, medical history and neurological examination findings are generally relied upon for diagnosis [9].

Studies have been conducted to evaluate the quality of YT videos that address various neurological disorders, such as arteriovenous malformations, narcolepsy, hydrocephalus, peripheral neuropathy, and infantile spasms. Studies have found that video content related to neurological disorders such as these is of low quality [4]. No research has of yet been conducted regarding the accuracy, adequacy, or usefulness of the information contained in YT videos about ET. The lack of data on the reliability of the information on this common movement disorder presented in videos uploaded to social media platforms such as YT reveals a clear need for study in this area. Therefore, we have aimed to evaluate the quality, reliability, and usefulness of ET-related videos on YT through the use of quantitative instruments. Our aim is to use the information uncovered in this work to inform readers of the highest-quality videos on the platform so that patients and their relatives as well as healthcare professionals may access this content more easily.

## Methods

### Data Collection

**Figure 1:**
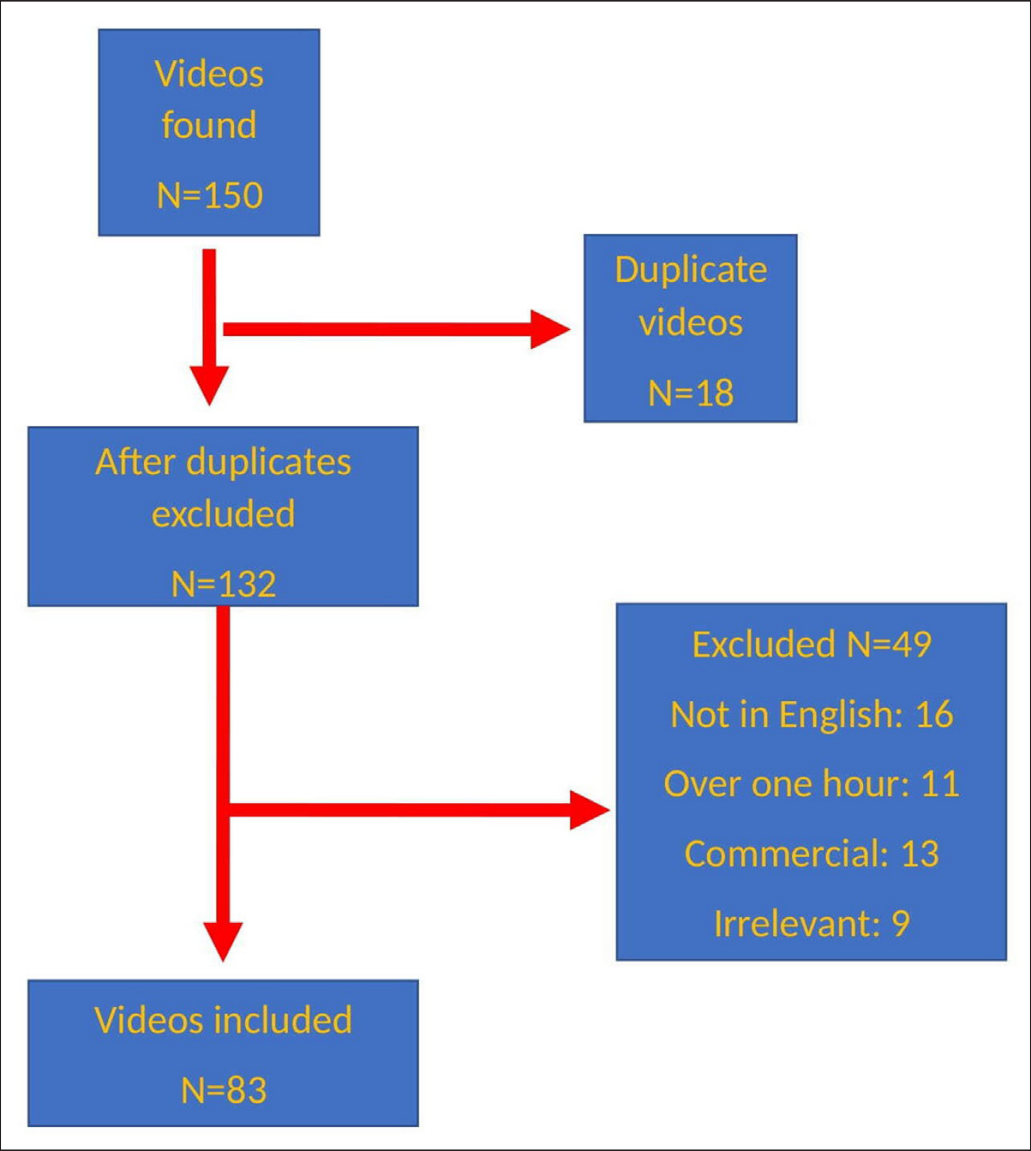
The flowchart of the selection of videos.

To perform the video analysis, we accessed YT using “incognito mode” on Google Chrome; to eliminate its potential effect on search results, we deleted the search history on the computer we used. We did not use a personal account to perform searches. We ran the searches using the default “relevance” “sort by” setting. On March 21, 2022, we searched for videos typing the keywords “essential tremor,” “postural tremor,” “action tremor,” “essential tremor hand,” and “essential tremor head” into the YT search bar. It has been reported that 90% of viewers do not move past the first 30 videos in search results; therefore we chose to review only the top 30 videos for each search term [10]. We imported the videos into a database before we viewed and analyzed them to determine the information a normal user would view and to obtain more objective data. Videos longer than 1 hour, those published in a language other than English, those without audio, duplicates, and irrelevant videos (e.g., commercial and advertising videos, music videos) were excluded from the study. A flowchart demonstrating the video exclusion criteria is provided in Figure 1. Ethics committee approval was not obtained, as in previous studies in the literature, since no animal or human data was used in the study and all videos were publicly available.

### Scoring System

The video content was evaluated by two independent practitioners, each of whom had 5 years of experience using the DISCERN criteria and the Global Quality Scale (GQS). DISCERN is a rating scale that has been designed to evaluate the quality and reliability of a publication or broadcast. The DISCERN scoring system comprises 16 questions on a scale from 1 to 5, with 1 point indicating that the quality criteria are not met and 5 points representing complete compliance with all quality criteria. Item 16 relates to a holistic and concise assessment of the entire video (see Table 1) [11]. To determine the final DISCERN score, the scores obtained from the first 15 questions are summed; the videos are classified as follows: excellent (63–75 points), good (51–62 points), fair (39–50 points), poor (27–38 points), and very poor (15–26 points) [12]. Scoring in GQS ranges from 1 to 5, with 1 to 2 points indicating low video quality, 3 points moderate quality, and 4 to 5 points high quality [13].

**Table 1:** DISCERN items and scoring.


QUESTION NUMBER	QUESTIONS	SCORING				

1	Are the aims clear?	1	2	3	4	5

2	Does it achieve its aims?	1	2	3	4	5

3	Is it relevant?	1	2	3	4	5

4	Is it clear what sources of information were used to compile the publication?	1	2	3	4	5

5	Is it clear when the information used or reported in the publication was produced?	1	2	3	4	5

6	Is it balanced and unbiased?	1	2	3	4	5

7	Does it provide details of additional sources of support and information?	1	2	3	4	5

8	Does it refer to areas of uncertainty?	1	2	3	4	5

9	Does it describe how each treatment works?	1	2	3	4	5

10	Does it describe the benefits of each treatment?	1	2	3	4	5

11	Does it describe the risks of each treatment?	1	2	3	4	5

12	Does it describe what would happen if no treatment is used?	1	2	3	4	5

13	Does it describe how the treatment choices affect overall quality of life?	1	2	3	4	5

14	Is it clear that there may be more than one possible treatment choice?	1	2	3	4	5

15	Does it provide support for shared decision-making?	1	2	3	4	5

16	Based on the answers to all of the above questions, rate the overall quality of the publication as a source of information about treatment choices.	1	2	3	4	5


### Audience Engagement Variables

We recorded quantitative data on video characteristics, including the total number of views, time elapsed since upload, video duration, number of comments, video power index (VPI), view ratio, like ratio, and number of likes and dislikes. VPI was calculated as (number of likes × number of views)/100, the view ratio was calculated as views after upload/days since upload, and the like ratio was calculated as number of likes/(number of likes + number of dislikes) × 100.

### Qualitative Variables

During the analysis, the practitioners noted whether the videos contained clear information concerning ET, such as symptoms, etiology, epidemiology, diagnosis, treatment, treatment response, prognosis, diagrams, radiological images, and animations; they also noted whether the presenter was a doctor or a patient. We classified the upload source of each video into one of five categories: doctor, hospital, education channel, patient, or other.

### Statistical Methods

We used the mean, median, range, and standard deviation descriptive statistics for the continuous variables and we applied the Mann-Whitney U test to evaluate the categorical variables. We used inter-rater agreement to determine the intra-class correlation coefficient and Spearman’s rank correlation coefficient was used to establish the correlation between the variables. Values that had a P of less than 0.05 were considered to be statistically significant. All analyses were performed using SPSS v. 21.

## Results

**Figure 2:**
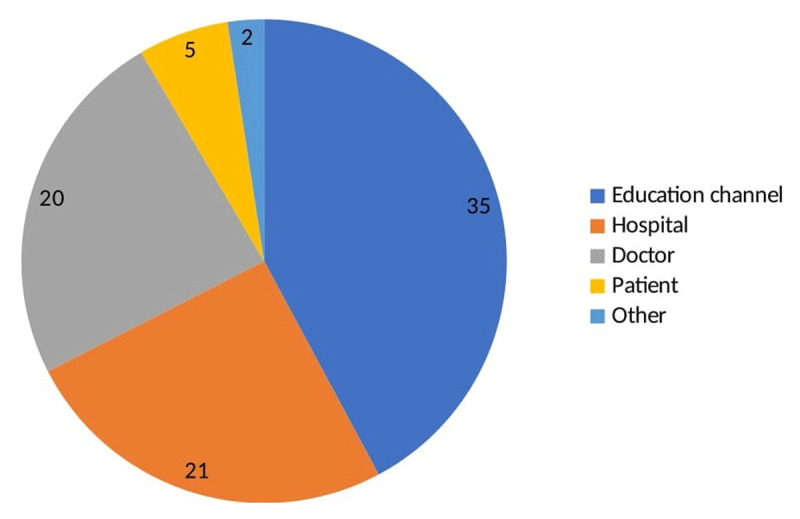
Source of uploaded videos.

Eighty-three videos met the inclusion criteria for the study. For the most part, the videos that were analyzed had been produced by education channels (42.2%, 35 videos). The distribution of the upload sources of the remaining videos was as follows: hospital, 21 videos (25.3%); doctor, 20 videos (24.1%); patient, five videos (6%); and other, two videos (2.4%). Data on the video sources are shown in Figure 2. In 60 of the videos (72.28%), the presenter was a doctor, while in 23 of the videos (27.72%), the presenter was a patient.

### Qualitative Video Content

**Figure 3:**
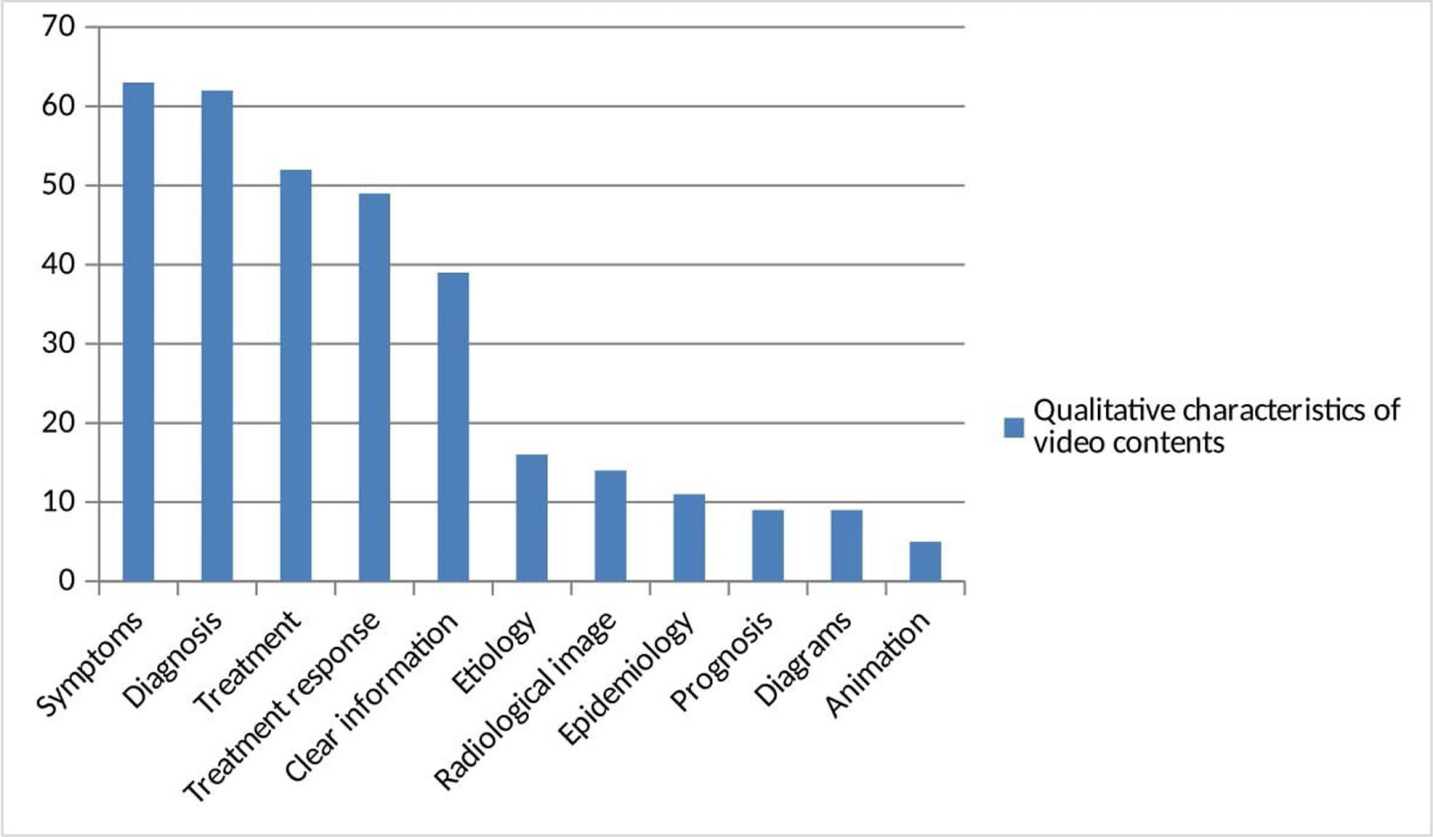
Qualitative characteristics of video contents.

While 75.9% (n = 63) of the videos addressed symptomatology, 74.7% (n = 62) actually provided diagnostic information. Videos that provided information about treatment and treatment response was determined to be 62.7% (n = 52) and 59.03% (n = 49), respectively, while those that provided clear information to viewers was 47% (n = 39). The proportion of videos that contained etiological and epidemiological data was 19.3% (n = 16) and 13.3% (n = 11), respectively. The qualitative characteristics of the content of the videos on ET are shown in Figure 3.

### Quantitative Video Content

The mean and range for the quantitative video metrics for all of the videos that were analyzed are as follows: number of views (mean, 48,634.21; range, 19–1,252,972), number of likes (mean, 327.10; range, 0–3,300), number of dislikes (mean, 12.46; range, 0–235), like ratio (mean, 91.34; range, 0–100), number of comments (mean, 54.97; range, 0–1,287), view ratio (mean, 28.08; range, 0.06–379.57), video duration (mean, 546.16 sec; range, 58–3,563 sec), number of days since upload (mean, 1,296.65; range, 25–3,530).

### Video Quality Metrics

**Figure 4:**
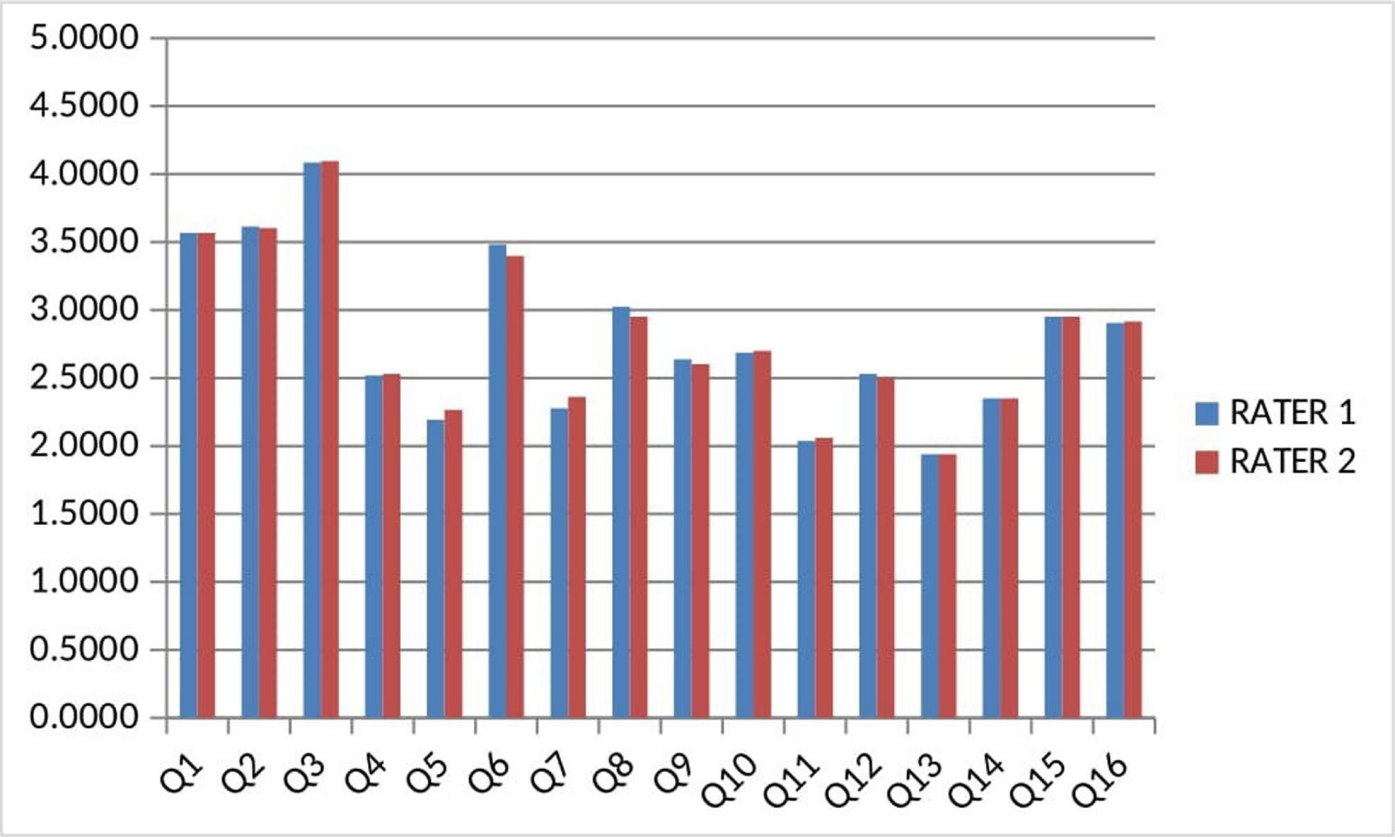
Mean DISCERN scores of the raters.

The mean DISCERN scores determined by the two raters were 41.95 ± 13.90 (range, 20–72) and 41.98 ± 14.04 (range, 19–72); the final mean DISCERN score was 41.96 ± 13.93 (range, 19–72). The mean GQS scores determined by the raters were 2.98 (range, 1–5) and 2.96 (range, 1–5). The mean DISCERN scores for item 16 were 2.90 (range, 1–5) and 2.91 (range, 1–5). The graph of DISCERN scores of both raters is shown in Figure 4. To ensure absolute agreement, we calculated the intra-class correlation coefficient between the two assessments; it was calculated as 0.9648 (95% confidence interval, 0.9468–0.9806) for single measures and 0.9344 (95% confidence interval, 0.9106–0.9602) for mean measures. This result indicates “excellent” reliability [14]. The mean DISCERN and GQS scores according to the video sources, education channel 42.82 and 3.05; hospital 50.19 and 3.71; doctor 38.10 and 2.75; patients 24.80 and 1.20; other sources were 21.50 and 1.22 respectively.

When we categorized the videos by quality classification based on their total DISCERN scores, 38.6% (n = 32) were in the poor category, 20.5% (n = 17) were in the fair category, 19.3% (n = 16) were in the good category, and 9.6% (n = 8) were in the excellent category. Further to this, the videos with ranked qualitative parameters had a significantly higher DISCERN score than those without: clear information (p < 0.001), symptoms (p = 0.045), etiology (p < 0.001), diagnosis (p = 0.001), treatment (p < 0.001), treatment response (p < 0.001), epidemiology (p < 0.001), presence of diagrams (p < 0.001), and presence of radiological images (p < 0.001). The videos that contained these elements also had significantly higher GQS scores than the remaining videos: clear information (p < 0.001), symptoms (p = 0.001), etiology (p < 0.001), diagnosis (p < 0.001), treatment (p < 0.001), treatment response (p = 0.001), epidemiology (p = 0.001), prognosis (p = 0.033), presence of diagrams (p < 0.001), and presence of radiological images (p < 0.001). The relationship between the average scale scores of the videos with and without qualitative data is shown in Table 2. Finally, the mean DISCERN and GQS scores for videos presented by a physician were significantly higher than for those presented by a patient (p < 0.001 for both). In the correlation analysis, we found a significant positive correlation between the DISCERN and GQS scores (p < 0.001, r: 0.907).

**Table 2:** Statistically significant relationships and selected qualitative video content.


	GROUP COUNT		MEAN DISCERN	STD	P	MEAN GQS	STD	P

**Clear information**	Yes No	39 44	49.30 35.43	2.21 1.56	**<0.001**	3.82 2.25	0.17 0.14	**<0.001**

**Symptoms**	Yes No	63 20	43.63 36.65	1.80 2.50	**0.045**	3.23 2.20	0.15 0.26	**0.001**

**Diagnosis**	Yes No	62 21	44.82 33.47	1.80 1.91	**0.001**	3.33 1.95	0.15 0.17	**<0.001**

**Treatment**	Yes No	52 31	48.01 31.77	1.83 1.38	**<0.001**	3.40 2.29	0.16 0.20	**<0.001**

**Treatment response**	Yes No	48 34	48.31 33.41	1.94 1.55	**<0.001**	3.39 2.44	0.17 0.21	**0.001**

**Etiology**	Yes No	16 67	56 38.59	3.43 1.43	**<0.001**	4.43 2.64	0.22 0.13	**<0.001**

**Epidemiology**	Yes No	11 72	57.72 39.54	4.25 1.44	**<0.001**	4.27 2.79	0.33 0.14	**0.001**

**Prognosis**	Yes No	9 74	52.11 40.71	5.59 1.52	0.050	3.88 2.87	0.42 0.14	**0.033**

**Diagram**	Yes No	9 74	60.44 39.70	4.27 1.43	**<0.001**	4.44 2.81	0.24 0.14	**<0.001**

**Radiological image**	Yes No	14 69	56.21 39.05	3.73 1.45	**<0.001**	4.35 2.71	0.22 0.14	**<0.001**

**Animation**	Yes No	5 78	50.80 41.38	7.90 1.53	0.213	4.00 2.92	0.54 0.14	0.076

**Doctor presenter**	Yes No	60 23	46.36 30.43	1.69 1.73	**<0.001**	3.43 1.82	0.14 0.18	**<0.001**


Mann-Whitney U test.

### Audience Engagement Parameters

Videos with ranked qualitative attributes had significantly higher audience engagement parameters than those without. The videos containing information on symptoms had significantly higher views (p = 0.034), while those containing information on etiology had a significantly higher like ratio (p = 0.023). We did not observe any other significant relationships between qualitative video features and audience engagement parameters, nor did we find a significant relationship between the presenter being a doctor or a patient and audience engagement parameters.

Between mean DISCERN scores and audience engagement parameters, we found the total number of views (p = 0.048, r: –0.217), view rate (p = 0.048, r: –0.217), number of comments (p = 0.017), r: –0.260), like ratio (p = 0.03, r: –0.239), and VPI (p = 0.032, r: –0.235) to be negatively correlated. There was also a significant negative correlation between the mean GQS score and the like ratio (p = 0.03, r: –0.239). The data of the correlation analysis are shown in Table 3.

**Table 3:** Correlation analysis.


	VPI	LIKE RATIO	TOTAL VIEWS	VIEW RATE	TOTAL COMMENTS	MEAN GQS

Mean DISCERN	–0.235*	–0.239*	–0.217*	–0.217*	–0.260*	0.907**

Mean GQS	–0.139	–0.239*	–0.121	–0.123	–0.194	1.000


Spearman’s rho. * Statistically significant at p < 0.05. ** Statistically significant at p < 0.01.

According to the DISCERN and GQS criteria, eight (9.6%) of the 83 analyzed videos were of excellent quality. The mean DISCERN score of these eight videos was 67.5, and their GQS score (for each of the eight videos) was 5. The mean VPI of these eight videos was 21.33. Table 4 presents further data on these eight top-quality videos.

**Table 4:** The Top eight Most Popular Essential Tremor Videos.


TITLE	UPLOADER	DISCERN	GQS	VPI	YOU TUBE LINK	DATE

Essential Tremor: Pediatric Onset	International Essential Tremor Foundation	68	5	4.91	https://www.youtube.com/watch?v=IU6Rhi6PUjA	10 May 2021

Diagnosis & Treatment Options for Essential Tremor	International Essential Tremor Foundation	72	5	49.28	https://www.youtube.com/watch?v=CJ8oZfme9wo	4 Aug 2020

TREMORS, Types, Causes, Treatment	Medi-Lectures	63	5	14.09	https://www.youtube.com/watch?v=o4QB6PNPTZo	29 Aug 2021

Latest Treatments For Essential Tremor and Parkinson’s Disease Webinar	Providence	70	5	3.46	https://www.youtube.com/watch?v=AcKdsxwxrEs	24 Mar 2021

Deep Brain Stimulation for Essential Tremor and Vocal Tremor	Stanford Health Care	63	5	3.78	https://www.youtube.com/watch?v=AV-2dNJczmI	1 Jun 2015

Treating Essential Tremor Today: From Propranolol to Piezoelectrics	Seattle Science Foundation	72	5	8.46	https://www.youtube.com/watch?v=zfOLHcWkc-I	1 Mar 2018

Essential Tremor and Thalamotomy	NEJM Group	68	5	2.96	https://www.youtube.com/watch?v=TOnoDGfq_kQ&t=1s	25 Aug 2016


## Discussion

In this study, we found that the overall quality and reliability of ET-related videos on YT was poor and almost half of the analyzed videos were in the poor and very poor category, which can be a detriment for patients who wish to gain medical insight and increase their knowledge about and control over their disease. Therefore, we consider that completely reliable information is not available to patients who seek information about ET on YT or for healthcare professionals who may want to use these videos for educational purposes. This research on this disorder that widely affects the global population is the first such study in the literature in the field of medical informatics.

Systematic review studies have revealed that there are many biased or incorrect health-related videos on YT [15]. One Canadian study reported that one-third of the adult population will use the internet to search for health-related information. It has also been found that more than one-third of those who visited a hospital due to a health problem discussed the information they encountered during their internet searches with their doctors [16]. Damage caused by misleading information can reach dangerous levels, and given that the majority of patients believe online information to be reliable, incorrect online medical information can lead to serious unforeseen problems [4,17].

A review of previous literature on YT content related to neurological disorders reveals that the content quality of YT videos about arteriovenous malformations, narcolepsy, and hydrocephalus was low [4]. On the other hand, one study reported that YT videos on stroke were of good quality according to the DISCERN criteria [18]. In addition, it has been reported that the accuracy rate of online information in studies where online content regarding conditions such as peripheral vertigo maneuvers, epilepsy, and movement disorders is analyzed varies between 30% and 60% [19,20,21]. Another study that examined Parkinson’s disease on YT revealed that only 19% of the videos were found to be useful or very useful [22]. The low quality of videos published on these neurological disorders, many of which affect a significant part of the global population and can have high mortality and morbidity, raises serious concerns.

Most of the videos in our study did not cite an information source or share when the information presented was produced, nor did they mention treatment risks, alternative treatment options, or how treatment might affect quality of life. However, we found that videos that did provide clear information contained information on symptomatology, treatment, response to treatment, and prognosis; contained etiological and epidemiological data; were enriched with diagrams and radiological images; and any animations were of higher quality in terms of content. Previous YT analysis studies on neurological diseases such as stroke and hydrocephalus have obtained similar results [18,23].

In this study, we observed that when videos with the highest VPIs were classified according to their mean DISCERN score, these videos fell into the “poor” and “very poor” categories. In addition, our correlation analysis uncovered a significant negative correlation between the mean DISCERN and GQS scores and audience engagement parameters (VPI, like ratio, etc.). These findings show that the most popular YT videos on ET are of low educational quality and may be misleading; therefore, using these videos for educational purposes could lead to shortcomings in viewers’ knowledge. The aim of those who upload videos with the highest VPIs may be motivated by commercial concerns to create content that will increase the number of views rather than to provide high-quality and essential information for the viewer. Studies have shown that videos that contain misleading content tend to achieve more audience engagement than videos that contain reliable content [24,25,26]. There are currently no obstacles in place that limit who can who can upload and share videos on YT. This may be one of the main reasons that many popular YT videos are of poor quality.

The first source of reference for education should be medical literature obtained from medical publications and guidelines. However, it is not always possible for a person who is not a healthcare professional to easily access scientific data. Furthermore, many people lack the search or evaluation skills necessary to access reliable information on online platforms. It is important, therefore, to raise the quality standards of medical videos uploaded to YT. Videos that contain quality educational content are undoubtedly more beneficial to patients seeking information and to the training of healthcare professionals, and they should theoretically receive more appreciation and interaction from viewers. As short, relevant videos are ranked higher by YT [27], content creators who aim to present accurate and useful data—such as hospitals, education channels, and doctors—should focus on producing short, concise, targeted videos that highlight important points, as this will enable them to reach a wider audience. Although the majority of the video uploaders in our study were educational channels and hospitals, and the majority of the narrators were doctors, the low quality of the content may be due to the competitive attitudes of doctors and medical channels that have become popular on social media. Popularity may have overtaken scientificity.

In this study, the two individuals who evaluated video quality were general practitioners (i.e., they did not specialize in neurology or movement disorders). Although this prevented bias, it may also have introduced a problem in terms of easy evaluation of some specific subjects (e.g., deep brain stimulation, radiological images, etc.). Another limitation of this research is that videos that were produced in languages besides English were not included in the study. Finally, we performed the searches at a single point in time; given that YT video content is constantly changing, this is an inevitable limitation that may affect all studies in this area.

In this study, we showed that the content quality of ET videos on YT is generally not high. While we did list the best-quality videos on ET as a reference for healthcare practitioners and patients, we do not suggest that medical videos should be used as a guide for diagnosis or treatment. It would be more appropriate for medical videos on YT to highlight informative features and direct followers to seek professional help. Patients should always verify information provided in online videos with more reliable sources of information. Since the world of the internet and YT are dynamic, it may be useful to repeat this study in a few years to assess whether there has been a change in the content quality of videos. Finally, those who seek to provide video content should ensure that it is optimized to attract the attention of viewers while presenting higher-quality information.
